# Supplementary material for: The Long-Jumping of African Swine Fever: First Genotype II Notified in Sardinia, Italy
Source: Viruses. 2023 Dec 23;16(1):32. doi: 10.3390/v16010032 (PMC10820622; doi:10.3390/v16010032)
Supplement: Supplementary file 1 [file viruses-16-00032-s001.zip › viruses-2706699-supplementary.pdf]

viruses

Supplementary Materials

The Long-Jumping of African Swine Fever: First Genotype II Notified in Sardinia, Italy

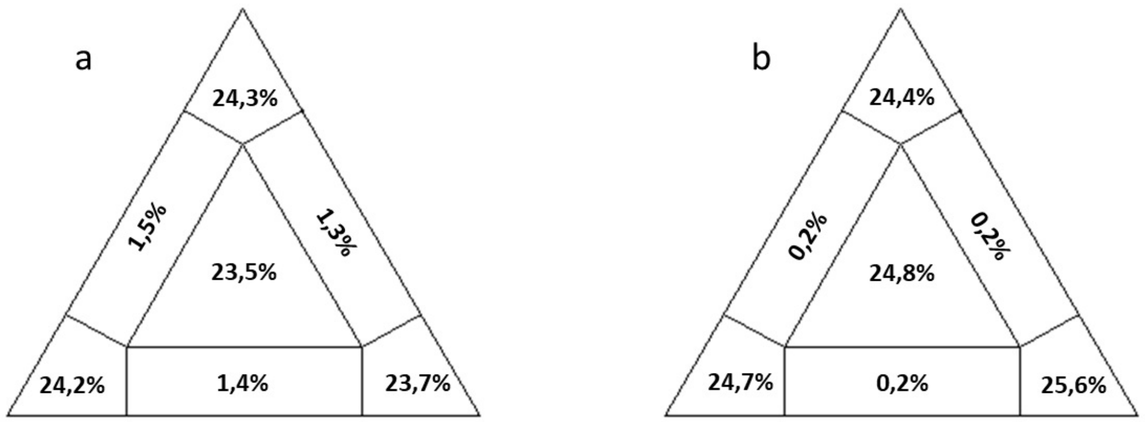

Figure S1. Phylogenetic signals of the data sets analysed in this study. a: Data set p72; b: data set p54.

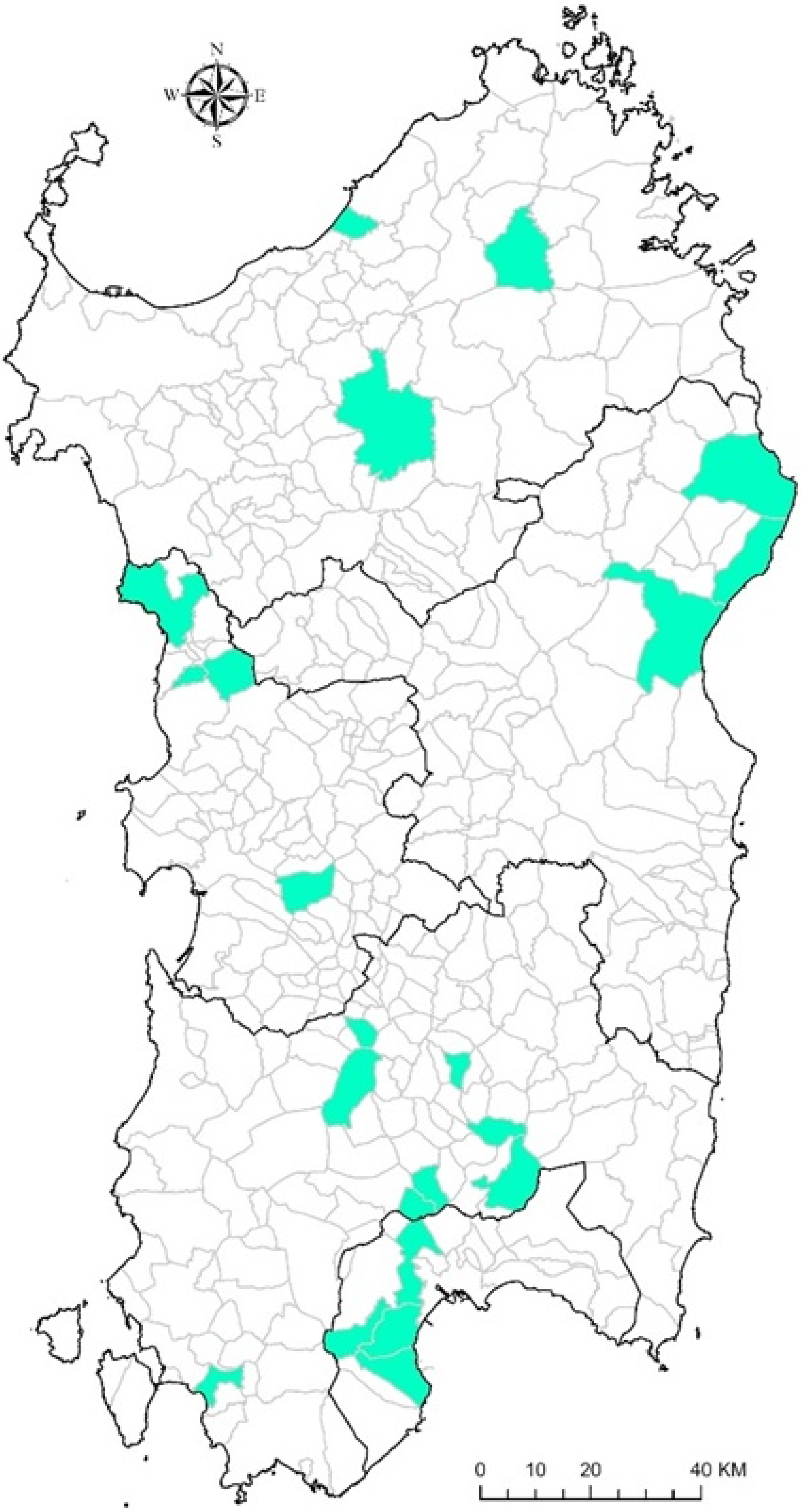

Figure S2. Sardinian municipalities where at least one retailer bought infected meat and meat products sold by Actor C2 and coming from Actor B.
